# High-Resolution Multiband 3D Imaging of Egyptian Papyri: Integrating Ultra-Close-Range Photogrammetry and Reflectance Transformation Imaging for Enhanced Documentation

**DOI:** 10.3390/s26072242

**Published:** 2026-04-04

**Authors:** Marco Gargano, Gianmarco Borghi, Eleonora Verni, Francesca Gaia Maiocchi, Sonia Antoniazzi, Viviana Goggi, Emanuela Grifoni

**Affiliations:** 1 Department of Physics Aldo Pontremoli, Università degli Studi di Milano, Via Celoria 16, 20123 Milano, Italy; 2 Marian Smoluchowski Institute of Physics, Faculty of Physics Astronomy and Applied Computer Science, Jagiellonian University, 11 Łojasiewicza St., 30-348 Kraków, Poland; gianmarco.borghi@doctoral.uj.edu.pl; 3 Doctoral School of Exact and Natural Sciences, Jagiellonian University, Ul. Prof. S. Łojasiewicza 11, 30-348 Kraków, Poland; 4 Department of Chemistry, University of Pavia, Via Taramelli 12, 27100 Pavia, Italy; eleonora.verni@unipv.it; 5 Arvedi Laboratory of Non-Invasive Diagnostics, Department of Musicology and Cultural Heritage, University of Pavia, Via Bell’Aspa 3, 26100 Cremona, Italy; 6 Fondazione Museo Egizio, Via Accademia delle Scienze 6, 10123 Torino, Italy; francescagaia.maiocchi@museoegizio.it; 7 Soseishi S.N.C. Restoration Laboratory, Via Cibrario 28, 10144 Torino, Italy; soanton@hotmail.com (S.A.); info@soseishi.com (V.G.); 8 Institute of Heritage Science, National Research Council of Italy, Via Madonna del Piano 10, 50019 Sesto Fiorentino, Italy; emanuela.grifoni@cnr.it

**Keywords:** papyrus documentation, multiband imaging, close-range photogrammetry, reflectance transformation imaging, cultural heritage 3D modeling, conservation science

## Abstract

Egyptian papyri are commonly documented using high-resolution two-dimensional imaging, which enhances legibility but does not adequately capture the micrometric surface morphology required for material and conservation studies. To address this limitation, we developed and validated an integrated, fully non-contact imaging workflow combining Ultra-Close-Range Multiband Photogrammetry with Reflectance Transformation Imaging (RTI) and normal map integration. The protocol was tested on six papyrus fragments from the Museo Egizio di Torino (XXI Dynasty–Byzantine period) exhibiting different conservation conditions. Multiband photogrammetry in the visible and visible-induced infrared luminescence bands achieved a Ground Sample Distance of 17 µm/px and a point cloud density of approximately 170 points/mm^2^, enabling detailed analysis of fiber morphology, surface deformation, and the spatial distribution of Egyptian blue. RTI-based normal map integration provided complementary high-frequency surface information with reduced acquisition and processing times. To overcome RTI low-frequency distortions, a revised normal integration strategy was implemented using surface planarization and frequency-domain fusion with photogrammetric data based on Power Spectral Density analysis. The resulting hybrid models combine metric reliability with enhanced surface detail, providing a scalable and non-invasive approach for papyrological documentation and conservation research.

## 1. Introduction

Historical documents written on paper, papyrus, or parchment are routinely digitized at high spatial resolution to enhance legibility and ensure effective long-term documentation and accessibility. In recent years, several major specialized projects have focused on the fundamental contribution of digital technologies, particularly computational imaging, to the study of textual materials and writing practices in ancient Egypt, emphasizing their relevance for epigraphical recording, papyrology, and scholarly dissemination [1,2,3]. However, these efforts have largely relied on two-dimensional imaging approaches that are inherently limited in capturing the micrometric morphological features of papyrus supports—features essential for materiality studies and informed conservation planning.

Three-dimensional imaging has already demonstrated significant diagnostic value for other quasi-planar heritage objects. In historical cartography, both laser scanning and close-range photogrammetry have been employed, with image-based approaches generally preferred for their ability to provide high-accuracy models while maintaining excellent texture fidelity and generating ortho-mosaics and Digital Elevation Models (DEMs) [4,5,6,7,8,9]. For panel paintings, 3D modeling has successfully monitored physical deformations of pictorial layers and support structures [10,11], enabled shape analysis during long-term restoration interventions [12], and facilitated advanced metrological analysis through multi-resolution models combining photogrammetric and conoscopic data [13]. RTI has also been applied to the study of panel painting surface details [14], the monitoring of conservation interventions [15], and the analysis of parchment and paper documents [16], as well as woodblock prints [17]. Pioneering studies have even integrated photogrammetry with multiband or hyperspectral data to identify pigments, reveal underdrawings and pentimenti, visualize paint alterations [18], and improve the readability of ancient papyrus scrolls [19]. While photogrammetry is widely used for physical acquisition, an alternative approach for creating digital twins, as illustrated by Chen et al. [20], combines 3D modeling with Generative Adversarial Networks (GANs) for the virtual restoration of garment heritages.

Despite these advances in related fields, papyri continue to be treated primarily as quasi-planar artifacts and are only rarely digitized in three dimensions. No comprehensive, systematic workflow has yet been established that integrates multiband imaging, ultra-close-range photogrammetry (UCRP) [21], and reflectance transform imaging (RTI) specifically for papyrus documentation. This gap stems from several methodological challenges: (1) the extreme fragility of papyrus requiring strict non-contact techniques; (2) the need for sub-millimeter resolution to capture fiber structure and surface microstratigraphy; (3) the requirement to preserve spectral information alongside geometric data; (4) practical museum constraints, including glass mounting and limited handling protocols. An integrated approach is particularly critical for papyri, where textual content, pictorial materials, and micrometric morphology are intrinsically interrelated and jointly contribute to both scholarly interpretation and conservation assessment.

This study addresses this gap by developing and validating an integrated workflow combining Ultra-Close-Range Multiband Photogrammetry with RTI-enhanced surface visualization. The specific contributions are: (1) a replicable multiband 3D imaging protocol for papyri; (2) quantitative comparison of photogrammetry versus RTI-based approaches for quasi-planar artifacts; (3) update of existing code for extracting meshes from RTI normal maps to incorporate a revised calculation method for mesh planarization; (4) demonstration of practical applications for papyrological research and conservation planning; (5) validation on six diverse papyrus fragments spanning multiple historical periods and conservation states.

The methodology integrates standard two-dimensional multiband imaging (visible reflected (VIS), near-infrared reflected (NIR), UV-induced visible fluorescence (UVF), and visible-induced infrared luminescence (VIL)) with two complementary 3D acquisition techniques: (1) ultra-close-range photogrammetry from RGB and VIL images; (2) Reflectance Transform Imaging coupled with normal map integration [22,23,24,25,26,27,28]. While RGB captures the visible appearance and VIL spatially resolves Egyptian blue distribution [29,30,31], the 3D techniques enable quantification of support texture, surface microtopography of pictorial films, and material losses at micrometer-level detail. The restoration of six papyri from the Museo Egizio di Torino—stored between glass plates with glued edges—provided an opportunity to validate this workflow and deliver morphometric data that enabled highly targeted conservation interventions.

## 2. Materials and Methods

The study was conducted on a corpus of six papyri undergoing complex conservation treatment necessitated by their fragile state of preservation. The fragments displayed issues primarily stemming from their conservation history; the intrinsic fragility of these artifacts was further compromised by restoration interventions carried out between the mid-nineteenth century and the 1970s. These earlier treatments employed techniques that are now recognized as inappropriate and have contributed to various forms of deterioration over time. Degradation is largely attributable to the use of synthetic adhesives, which have progressively altered the chemical and structural stability of papyrus, affecting both its elasticity and chromatic characteristics. To inform the conservation process, several analyses were conducted, including imaging investigations, which are the focus of the present study.

### 2.1. Case Studies: Papyri from the Museo Egizio Collection

The six fragments are presented in Table 1 and differ significantly in composition and fabrication, reflecting their diverse functions and chronological origins. Variations are evident in size, structure (with kollesis present in Cat. 2117), form (e.g., Cat. 2117, Cat. 1975), the presence of edge cutting (e.g., Cat. 2117), writing systems, and graphic media. For instance, fragments Suppl. 6101 and Cat. 1975 display distinct modes of graphic media application, as writing marks are visible on both sides of the papyrus support. With the exception of the fragment CP103 SN, which was mounted between plates of extra-clear safety glass, all papyri were previously displayed between two sheets of common industrial glass prior to restoration. The mounts were sealed using various materials, including gummed paper and pressure-sensitive tapes. Additionally, the fragments were affixed to the glass with different adhesives and support materials—such as bovine peritoneum, Japanese tissue, medical gauze, and imitation parchment (pergamyn)—and featured either partial (Cat. 1975) or total (CP103 SN) linings [32,33].

The proposed methodologies were applied to all the papyrus fragments examined in this study. For clarity and brevity, however, the results are mainly illustrated through the detailed discussion of a single representative fragment, chosen because it contains materials that show detectable responses both in visible imaging and in VIL.

### 2.2. High-Resolution Multiband Imaging

**Figure 1:**
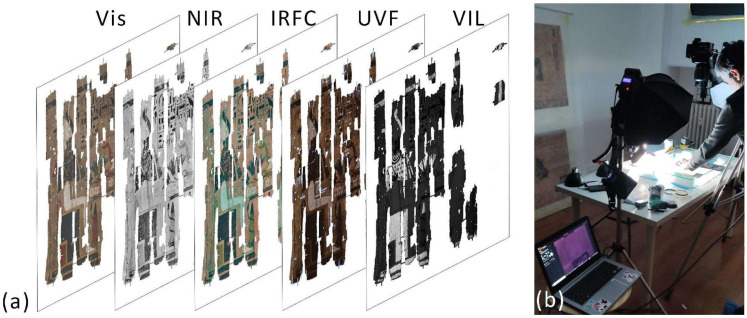
(**a**) Visualization of the multiband cube (VIS, NRR, IRFC, UVF, VIL images) of the Provv. 6255 papyrus; (**b**) setup for the multiband acquisitions.

The six papyri that were examined and restored are part of one of the world’s greatest collections, the Museo Egizio in Turin, which houses some 17,000 fragments. Each papyrus was analyzed in different spectral bands using the Phase One XF camera system (Copenaghen, Denmark), equipped with a digital IQ3 back, a “Trichomatic” CMOS detector (100 MP) without an IR-cut off filter to extend the spectral sensitivity to 1000 nm, and a Schneider Kreuznach 120 mm Macro LS f/4 lens (Bad Kreuznach, Germany). For the VIS and NIR acquisitions, an illumination source consisting of two Godox Witstro 360 W flashes was used (GODOX Photo Equipment Co., Ltd. Shenzhen, China). A B+W 093 (Schneider Group Germany, Bad Kreuznach, Germany) band-pass filter set between 850 and 1000 nm was placed in front of the lens to capture NIR photos. Two 3 W LED lamps with a peak emission wavelength of 365 nm ± 10 nm were utilized to capture UVF pictures, and a KV 418 filter that cuts-on at 420 nm was placed in front of the lens to remove the visible blue-violet parasitic component present in the LED emission. For VIL imaging, two 35.2 W DORR LED DLP-600 lamps and an 850–1000 nm bandpass filter were used. The papyri were analyzed at the Soseishi Restoration Laboratory in Turin, to which they were transferred from the Museo Egizio for conservation treatment. Each papyrus was placed horizontally on a glass surface and illuminated with lamps positioned symmetrically at 45°, as shown in Figure 1b. The acquisition and post-processing of the image sets (VIS, NIR, IRFC, UVF, and VIL) produced a high-resolution multiband data cube for each papyrus (Figure 1a).

### 2.3. Reflectance Transformation Imaging (RTI)

Reflectance Transformation Imaging (RTI) is a technique used to estimate the intensity and direction of light reflected from an object, enabling its visualization under varying directions of incident illumination [34]. This approach has rapidly become a widely adopted tool for the documentation, acquisition, and analysis of cultural heritage objects, as it enhances surface details and reveals features that are otherwise difficult to detect [35,36]. The outcome of this mathematical synthesis of illumination information is an RGB pseudo-color image, commonly referred to as a normal map.

**Figure 2:**
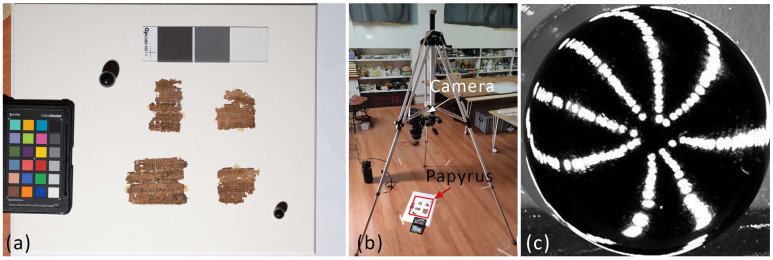
RTI acquisition set-up showing (**a**) the Provv. 8571 and F 399/2 papyri with black reflective spheres, a ColorChecker chart, and metric references; (**b**) position of the camera and the papyrus during the acquisitions; (**c**) highlight images on one of the two reference spheres showing the lighting positions.

RTI images were also captured with the Phase One XF camera system, mounting a 120 mm macro lens. The camera was positioned to frame the papyrus fragment from a top-down perspective, with the papyrus placed on the floor (Figure 1b). For lighting, a Godox Witstro 360 W flash was manually positioned according to different lighting directions; two black reflective spheres together with a ColorChecker Passport Photo served as references (Figure 2a). Ninety-five photographs were acquired while maintaining a constant sensor–object distance of 65 cm, each corresponding to a different lighting position. The distribution of the light sources on the black reflective sphere is shown in Figure 2c; despite manual positioning, a homogeneous illumination was achieved. The asymmetry observed in the lower-left portion of the sphere was caused by obstruction from a tripod leg. The RTI images were acquired using Relight 1.3.1 software [37], and they were visualized using RTI Viewer 1.1 software [38].

Normal integration was subsequently applied to the estimated normal map, resulting in the reconstruction of the corresponding three-dimensional surface. A global integration approach was adopted, based on the Frankot–Chellappa algorithm [39]. Specifically, surface reconstruction was achieved using the global least-squares (L2) solver implemented via the Fast Fourier Transform (FFT) developed by Quéau et al. [40].

### 2.4. Ultra-Close-Range Multiband Photogrammetry

**Figure 3:**
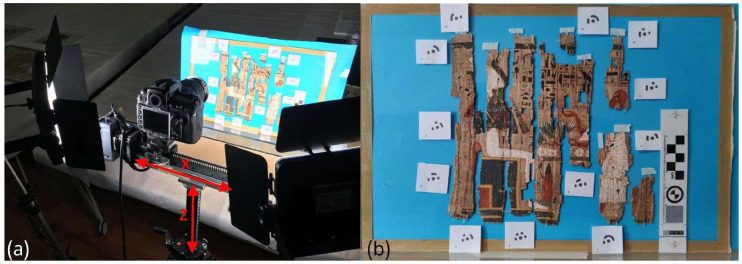
(**a**) VIS and VIL ultra-close-range photogrammetric set-up with the camera mounted on the motorized linear stage; (**b**) the position of papyrus Provv. 6255 during 3D macro acquisitions with coded target and metric reference.

For the VIS and VIL ultra-close-range photogrammetric acquisition (referring from now on to the Provv. 6255 papyrus), a Nikon D810 (Nikon corporation, Minato, Tokyo) full-frame digital SLR camera with a 36 Mpixel FX CMOS sensor was used, on which a Micro Nikkor 105 mm f/2.8 fixed focal length lens (Nikon corporation, Minato, Tokyo) was mounted. Two DORR LED DLP-600 lights of 35.2 W each were used as visible light sources for both the bands considered (Figure 3a). To obtain a correct chromatic representation, a ColorChecker Passport Photo (Calibrite LLC, Wilmington, USA) was used, while to achieve an accurate metric restitution of the 3D model, 12 scaling targets and corresponding scale bars were placed all around the edges of the papyrus (Figure 3b). The camera was mounted on a Manfrotto tripod with the sensor plane in a horizontal position, parallel to the papyrus, to optimize the field of view for optimal utilization of the frame. A motorized linear stage, approximately 20 cm in length, was mounted on the tripod’s vertical rack column, allowing for motorized movement along the X (horizontal) axis, while vertical (Z) adjustments were performed manually using the tripod column (Figure 3a). The sensor-to-object distance was maintained at 37.5 cm, enabling scanning of the entire surface of the papyrus.

**Figure 4:**
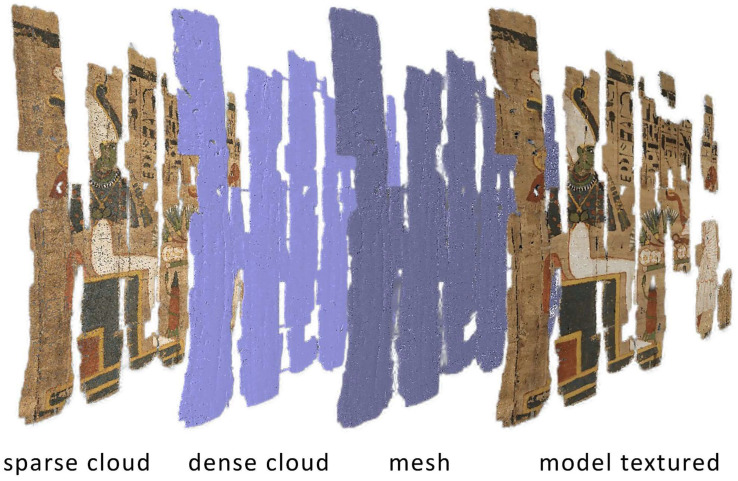
Workflow of the photogrammetric processing performed in Agisoft Metashape Professional, showing the main reconstruction steps from image alignment and sparse point cloud generation to dense point cloud computation, mesh creation, and final textured 3D model.

After image acquisition, the collected dataset was processed using the commercial photogrammetric software Agisoft Metashape Professional 2.2.3. The workflow followed the standard Structure-from-Motion (SfM) and Multi-View Stereo (MVS) pipeline implemented in the software. Initially, the images were aligned to estimate camera positions and orientations, resulting in the generation of a sparse point cloud representing the main geometric structure of the scene. Subsequently, a dense point cloud was computed from the aligned images to provide a more detailed representation of the object. This dense reconstruction was then used to generate a polygonal mesh, which defines the surface geometry. Finally, a high-resolution texture was projected onto the mesh to obtain the fully textured 3D model. The main processing steps and the resulting model are illustrated in Figure 4.

**Figure 5:**
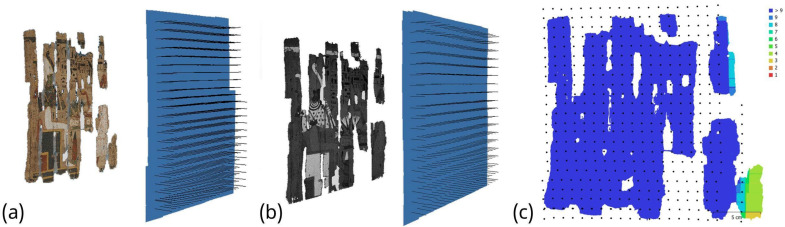
Camera positions for the (**a**) VIS and (**b**) VIL 3D modeling of the Provv. 6255 papyrus using the scanning method on a motorized linear stage; (**c**) camera locations and image overlap where most of the surface of the model corresponds to an overlap of more than nine contiguous images.

Each 3D spectral model has a total of 464 photos captured automatically per centimeter with a 90% overlap along both axes in this setup (Figure 5a–c). Since depth of field is important in macrophotography, a fixed aperture of f/32 was used to take shots that were all in focus and avoid the time-consuming focus-stacking procedure. As a result, the height difference was about 0.85 mm between the front and back of the focused spot, i.e., within the expected focal depth of field.

## 3. Results and Discussions

### 3.1. Ultra-Close-Range Multiband Photogrammetry Metrological Assessment and Diagnostic Potential

The photogrammetric processing of the 464 Vis and VIL images produced high-density 3D spectral models exported in .OBJ and .PLY formats. To preserve the qualitative integrity of the point clouds and meshes required for micrometric morphological analysis, no data simplification or resampling was applied [41]. Quality assessment focused on three key parameters: point cloud density, geometric resolution, and theoretical coordinate accuracy. Point cloud density is a primary indicator of 3D model accuracy, as adequate spatial sampling is essential to faithfully represent the geometry of the object [42,43,44]. Density was calculated using CloudCompare’s dedicated tool, which calculates the average number of nearby points within a radius of 0.01 m for each point and proportionally estimates the surface density across the entire cloud. The resulting dense point clouds achieved a surface density of approximately 170 points/mm^2^. This high density ensures that fine-scale features, including individual papyrus fibers (typical width 100–300 µm) and ink deposits, are adequately sampled for subsequent morphometric analysis. The accuracy of coordinates in Structure-from-Motion (SfM) photogrammetry depends crucially on the accuracy of the extraction and matching of homologous points in the image space, which typically achieves sub-pixel accuracy [45,46]. Assuming a conservative estimate of 0.5 pixels of accuracy in image space (corresponding to 2.43 µm given the pixel pitch of 4.87 µm), the theoretical accuracy of the coordinates can be estimated from the geometric configuration and GSD [47,48,49,50]. For our acquisition configuration, the propagated uncertainties result in: XY plane accuracy: ±8.6 µm; *Z*-axis accuracy: ±326 µm. The combination of high point cloud density (170 points/mm^2^) and fine geometric resolution (17 µm/px) allows for quantitative analysis of the materiality of papyrus at scales previously only achievable with expensive optical profilometry systems. Specifically, this level of detail can support: (1) The analysis of the fiber structure: It is possible to resolve the orientation of individual fibers and weaving patterns. (2) The characterization of pigments and ink: Three-dimensional profiles of pigment and ink deposits allow for the distinction of morphological characteristics of the color areas, writing instruments and application techniques. (3) The assessment of the state of conservation: It is possible to quantitatively map material losses, errors in the positioning of papyrus fragments, surface deformations, and adhesive residues.

### 3.2. Orthomosaics and Digital Elevation Models

The photogrammetric workflow generated two complementary metric products from the dense point clouds: high-resolution orthomosaics and Digital Elevation Models (DEMs), which are essential for long-term conservation documentation. In fact, orthomosaics serve as a unified reference layer for integrating diagnostic data from multiple sources, as they provide a planimetrically correct representation in which distances, areas, and angles can be measured directly. This capability has proven particularly valuable for mapping the distribution of materials and documenting areas of loss, adhesive residues, and previous restoration interventions. One potential application could be the co-registration of pre- and post-restoration orthomosaics to detect changes with micrometric precision.

In the case of the papyri under examination, the orthomosaics were generated through the process of orthorectification and mosaicking of the input multiband images, maintaining radiometric fidelity and thus allowing for precise spatial correlation of the distribution of materials with morphological characteristics.

**Figure 6:**
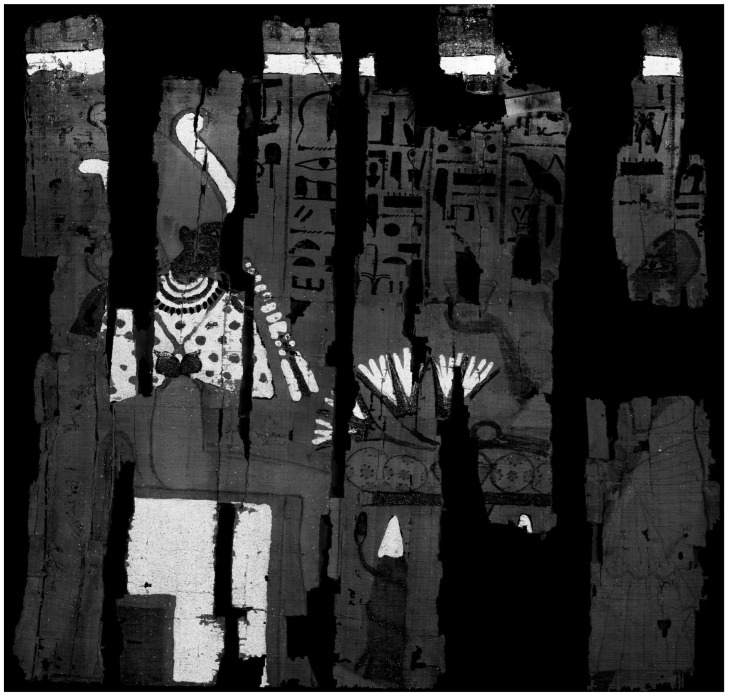
Orthomosaics in the VIL band.

**Figure 7:**
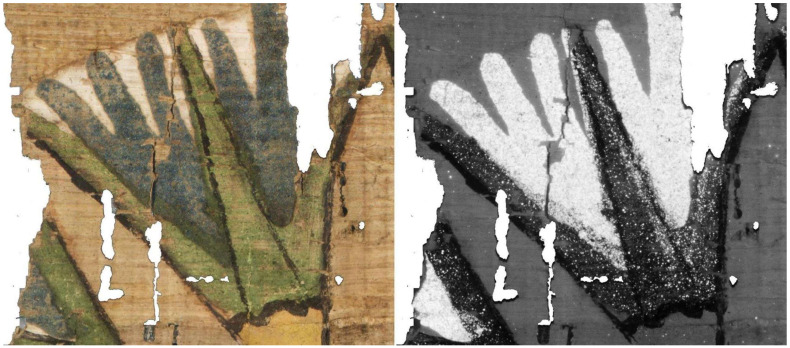
Comparison of VIS and VIL orthomosaic details in the area of the lotus flower, where Egyptian blue pigment grains are visible in both the blue and green regions.

For each papyrus, the multiband orthomosaics achieved a total resolution of 1.07 gigapixels (Gpx), with a pixel size of 8.2 µm/px, approximately twice the spatial resolution of the photogrammetric dataset (GSD = 17 µm/px). The orthomosaic obtained in VIL is shown in Figure 6, while Figure 7 presents a comparison between VIS and VIL orthomosaic details in the area of the lotus flower. The grains of Egyptian blue pigment, in addition to being clearly visible in the blue areas, where their presence is expected, can also be observed within the green painted regions. It can be reasonably assumed that the artist used blue pigment to enrich the chromatic appearance of the green leaves and may also have employed the same brush for both blue and green pigments, resulting in residual grains of Egyptian blue within the green areas.

The DEMs were generated by projecting the dense 3D point cloud onto the XY reference plane, with each raster cell encoding the Z coordinate (elevation) of the papyrus surface. This representation transforms the complex 3D point cloud into an easily analyzable 2.5D format in which elevation information can be queried, visualized, and quantitatively processed. In our process, the DEMs derived from photogrammetry achieved a total resolution of 1.56 gigapixels (Gpx), with a theoretical pixel size of 8.2 µm/px, based on photogrammetric geometry.

The following section describes the complementary morphometric information provided by the integration of photogrammetry and RTI.

### 3.3. RTI-Photogrammetry Integration: Technical Implementation

**Figure 8:**
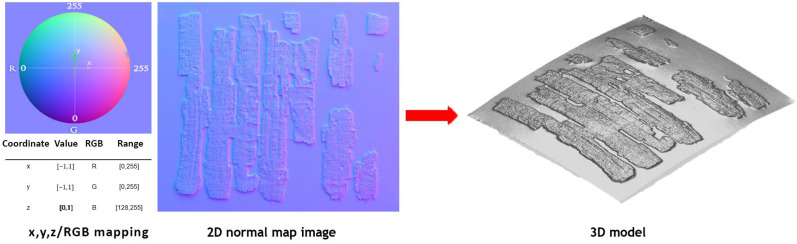
From 2D to 3D: A 2D RTI normal map image gives a 3D RTI model after integration.

Starting from the normal map obtained through the RTI acquisition, it is possible to reconstruct the surface profile of semi-flat objects by integrating the normal vectors derived from RTI, a process known as normal integration [40,51], as schematically illustrated in Figure 8.

The normal integration technique has been known for many years and has been widely employed in computer graphics and 3D imaging for surface reconstruction and modeling. In the field of cultural heritage, this approach has already been applied to the reconstruction of semi-flat objects, although with known limitations in the accurate representation of low-spatial-frequency components of the surface geometry [52,53,54].

**Figure 9:**
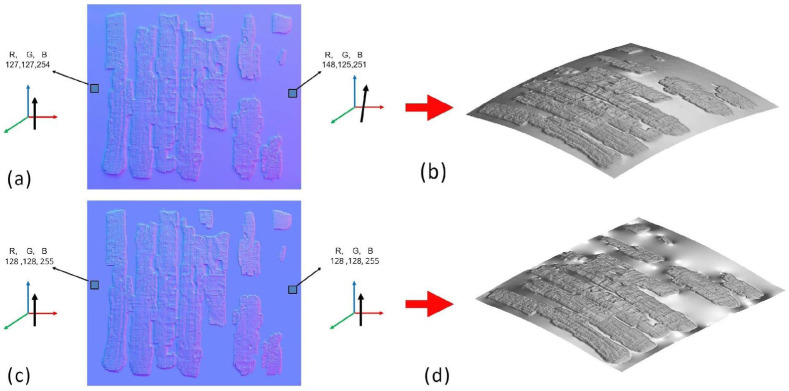
3D reconstructions starting from (**a**) the original normal map and (**c**) the recolored normal map, giving the (**b**) and (**d**) 3D reconstructions of the papyrus, respectively. The direction of the arrows at the sides of the (**a**,**b**) images indicates the orientation of the surface normals in the highlighted black squares.

To combine the metric and geometric information derived from photogrammetry with the higher spatial resolution provided by RTI, it is necessary to correct distortions, scale the object to its true dimensions, and assign the photogrammetric coordinate system to the reconstructed surface. This enables the RTI-derived model to be reintegrated into the photogrammetric workflow (e.g., re-imported into Metashape for texturing). Two different methods were tested to achieve this integration. One of the main sources of error introduced by the normal integration process is the warping of the base plane. Although the illumination points during the RTI acquisitions were distributed uniformly, small differences in flash intensity or positioning can still introduce artifacts. This results in a surface that should be flat appearing deformed after the normal integration procedure. This effect can be verified by measuring the RGB coordinates along the edges of the plane, which should be uniform but instead differ by approximately 15%. In a first approximation, this artifact can be corrected using a mask that sets the background to the same RGB coordinates (128, 128, 255), representing a surface with a perfectly vertical normal vector as illustrated in Figure 9.

**Figure 10:**
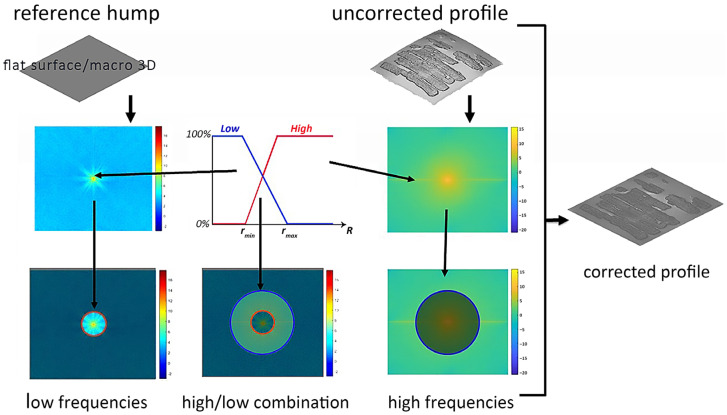
Scheme of the correction applied to the model obtained through normal map integration: the high spatial frequencies of the uncorrected model are mixed with the low frequencies of a flat reference surface or a 3D render obtained through low-resolution photogrammetry.

Another approach to flatten the base plane of the deformed 3D model consists of generating a reference flat surface and combining its low-spatial-frequency components with the high-spatial-frequency information provided by the RTI technique, as schematically illustrated in Figure 10.

**Figure 11:**
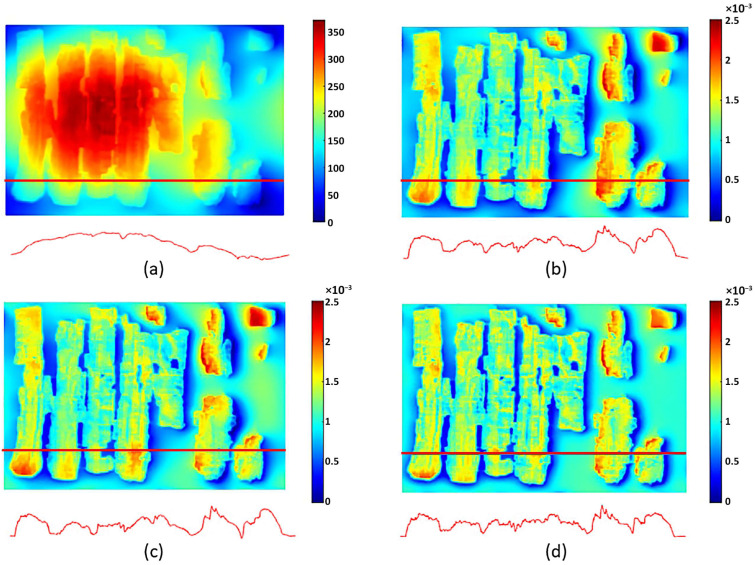
(**a**) Height profile of the 3D model obtained by RTI normal map integration, compared with the height profiles of the same model combined with a flat reference surface using different parameter ranges: (**b**) r_min_ = 1 and r_max_ = 5, (**c**) r_min_ = 2 and r_max_ = 5, and (**d**) r_min_ = 1 and r_max_ = 10. The red curves below each height profile represent the line profiles corresponding to the red lines overlaid on the plot.

The method employed was proposed in [52]. Starting from the code of [40], additional implementations were introduced. The approach involves calculating the Power Spectral Density (PSD), a fundamental tool in signal processing that characterizes the distribution of a signal’s frequency components by quantifying the contribution of each frequency to the total signal power. Using the PSD of the artificial flat surface makes it possible to select its low spectral frequencies (PSD_low_). Afterward, the PSD of the high frequencies of the object is calculated from the normal map (PSD_high_), and the two PSDs are combined as shown in Equation (1):

(1) $$PSD_{new}\;=\;\alpha PSD_{low}\;+\;\left(1\;-\;\alpha \right)PSD_{high}N$$

parameter N is a normalization factor, and parameter α, displayed in Equation (2), governs the transition from low to high frequencies:

(2) $$\alpha \;=\;1\;-\;\frac{\left(R-r_{min}\right)}{\left(r_{max}-r_{min}\right)}$$

where r_min_ represents the lower spatial frequency threshold (or radial cutoff frequency). It identifies the specific radius in the frequency domain that marks the beginning of the transition zone. Mathematically, it is the boundary separating the lowest frequencies, which defines the macroscopic shape of the surface, from the frequency band where the mixing process with the flat surface’s PSD actually begins. A small value of r_min_ prevents the process of mixing the frequencies. Increasing the value of r_min_ brings the real surface closer to the flat one, although the real low frequency is brought to zero by significantly increasing the value of r_min_. Increasing the value of r_max_ also makes the real surface more similar to the flat surface, as it gives more weight to low frequencies. A large difference between r_min_ and r_max_ results in a slower transition between the two PSDs. The height profile of the 3D model obtained by RTI normal map integration, compared with the height profiles of the same model combined with a flat reference surface using different combinations of r_min_ and r_max_ is presented in Figure 11 together with a line profile (in red) that plots the variations in height along a line.

**Figure 12:**
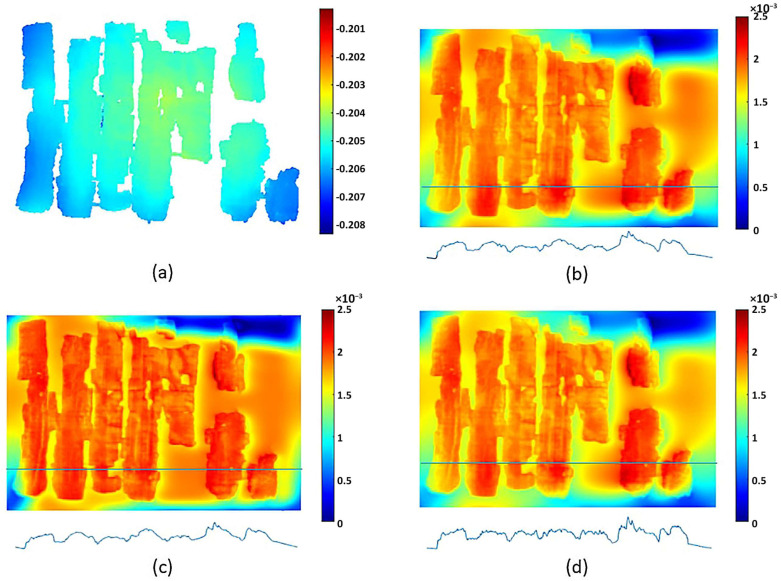
(**a**) Height profile of the simplified 3D model obtained by ultra-close-range photogrammetry, compared with the height profiles of the same model combined with the 3D model derived from RTI normal map integration using different parameter ranges: (**b**) r_min_ = 1 and r_max_ = 5, (**c**) r_min_ = 2 and r_max_ = 5 and (**d**) r_min_ = 1 and r_max_ = 10. The blue curves below each height profile represent the line profiles corresponding to the blue lines overlaid on the plot.

The same method can be applied to combine the high-frequency information derived from RTI with the low-frequency components obtained from a simplified point cloud generated by ultra-close-range photogrammetry in the Vis range. Figure 12 illustrates the differences between the height profiles resulting from the combination of photogrammetry and RTI normal map integration using different r_min_ and rmax parameter values.

The selection of r_min_ and r_max_ is based on the sensor-specific noise floors and on how reliable the geometric information is at different spatial frequencies. The parameter r_min_ is set to the frequency where the low-frequency bias (artifacts) of the normal integration becomes non-negligible. Frequencies lower than 1/L (where L is the papyrus fragment length) are dominated by integration artifacts, rather than actual topography given by the RTI. By setting r_min_ between 1 and 2, the RTI topography is anchored to the absolute spatial coordinates provided by the flat surface or the photogrammetry.

Regarding the parameter r_max_, which defines the limit of the transition zone, a value of r_max_ = 5 (as seen in Figure 11b,c) represents a narrow transition band, while a value of r_max_ = 10 (as seen in Figure 11d and Figure 12d) provides a gradual frequency blending. This wider transition is preferred because it reduces aliasing and artifacts that can appear when combining two datasets with different characteristics. Depending on the specific requirements of the study, it may be more advantageous to integrate the surface obtained with photogrammetry rather than the artificial flat surface. Regardless of the method chosen, RTI and normal integration techniques provide high accuracy for high spatial frequencies, potentially up to the Nyquist frequency [55]. The Nyquist frequency defines the theoretical resolution limit imposed by the digital model’s sampling density (or pixel pitch). Mathematically expressed as half the sampling rate (f_N = 1/2∙f_Sample), it serves as a threshold for detail preservation. Consequently, stating that the integration techniques maintain accuracy up to this limit implies that the method fully exploits the discretization grid, successfully reconstructing the finest surface features physically allowed by the sensor’s resolution.

To clarify its structure and functioning, the computational architecture of the developed MATLAB R2024a code is organized into four sequential processing modules:

1. Surface Integration: The system first converts the RTI normal maps into gradient fields (*p*, *q*) and performs a fast least-squares integration using a Poisson solver with Fast Fourier Transform (FFT) to generate a high-frequency depth map [40].
2. Frequency Domain Fusion: The low-frequency photogrammetric point cloud is scaled and interpolated onto the 2D image grid. The software computes the Power Spectral Density (PSD) for both the integrated high-frequency surface and the low-frequency reference using 2D FFT. These are blended in the frequency domain governed by user-defined radial thresholds (r_min_ and r_max_), and reconstructed into a single surface via inverse FFT.
3. Background Masking: Undesired background areas are removed by mapping the combined 3D coordinates against a 2D binary mask.
4. Z-Preserving Alignment: The final point cloud is aligned to the object-space defined in Metashape using a customized registration algorithm.

Detailing the post-processing phases, the background removal employs a mask-based approach. The process begins by reading a text file containing the three-dimensional coordinates (X, Y, Z) of the combined RTI point cloud. Simultaneously, a binary image is loaded as a mask that defines which areas of the point cloud to retain and which to discard. For each point, the software checks the corresponding pixel in the mask: if the pixel is black, the software zeroes the point’s Z coordinate. This zeroing step flags points for removal. Subsequently, all points with a zero Z coordinate are eliminated, completing the background-cleaning operation.

The final operation, point cloud alignment, is more complex and involves multiple steps to align the model within the photogrammetric object-space. This method utilizes a combination of nearest neighbor searches (k-NN) [56] and Iterative Closest Point (ICP) [57] algorithms. Prior to alignment, an initial scale estimation is performed based on the 2D bounding boxes of the point clouds. This normalization is a temporary step that preserves the object’s true dimensions while performing distance comparisons. The k-NN algorithm then establishes initial correspondences between the two datasets, and the ICP iteratively refines these matches—calculating 2D rotation, translation, and scale—to achieve optimal alignment. A defining feature of this software is its preservation of the RTI cloud’s original Z values during alignment. Depth and height values deriving from RTI often exceed the accuracy of those deriving from photogrammetric measurements; therefore, the algorithm isolates and operates solely on the X and Y coordinates, fully retaining the original depth information for the final output.

Finally, a minor manual adjustment, when needed, can be performed in CloudCompare. Since Z values are excluded during alignment, a slight shift may occur between the object and the aligned cloud. This last refinement is rapid, thanks to the preceding preprocessing steps.

Through this workflow, we can generate a highly informative point cloud that integrates the high-frequency details from RTI with the low-frequency components of photogrammetry. This process creates a scaled 3D model suitable for metric calculations. Furthermore, by aligning the generated model within the same object space as the photogrammetric reference, it can seamlessly replace the original geometry within the processing software to be fully textured.

**Figure 13:**
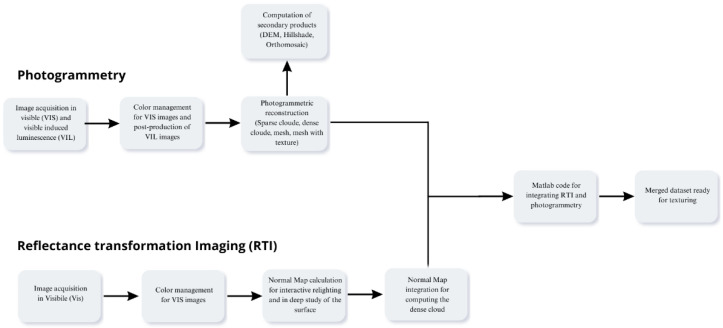
Workflow of the RTI and photogrammetric integration used in this research.

The complete custom MATLAB script developed for this pipeline is available at [58] to ensure full transparency and reproducibility of the described computational architecture. Figure 13 schematically shows the workflow of the methodology used.

### 3.4. RTI and Multiband Photogrammetry: Texture Comparison

An important aspect in the comparison of the different methods employed concerns the results that can be obtained and used to assess the conservation state of the papyrus and to identify the materials present on its surface. This section, therefore, presents a comparison of selected details highlighting the strengths and limitations of the different approaches in the specific case of a papyrus that, in addition to the text, also contains pictorial elements.

**Figure 14:**
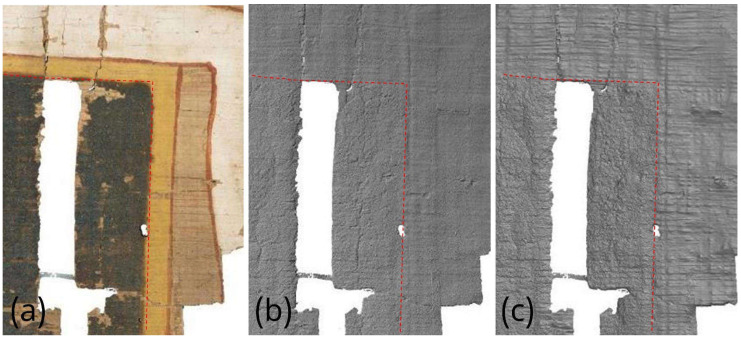
Comparison of the morphological representation of a papyrus detail obtained using: (**a**) visible-light image (VIS); (**b**) VIS ultra-close-range photogrammetry; (**c**) processed RTI normal map with flattened base plane. The dotted red lines define the area corresponding to the Egyptian blue.

**Figure 15:**
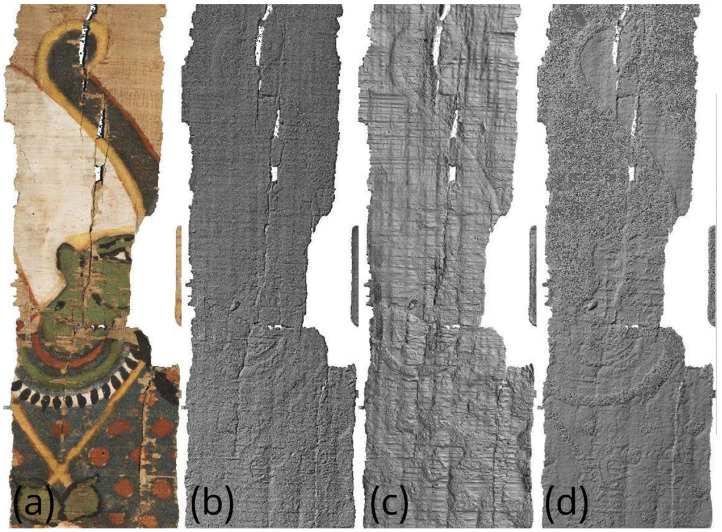
Comparison of the morphological representation of a papyrus detail obtained using: (**a**) visible-light image (VIS); (**b**) VIS ultra-close-range photogrammetry; (**c**) processed RTI normal map with flattened base plane; (**d**) VIL ultra-close-range photogrammetry.

To better compare a particularly interesting portion of these artifacts, it is appropriate to analyze their surface morphology in detail, neglecting the global deformation discussed in the previous section, as only very limited-sized details are considered here. For the comparison, in addition to the visible-light image, Digital Elevation Models are used for VIS and VIL photogrammetry, while for RTI, normal map processing is employed, where the RGB image is converted into a grayscale height-profile image. Starting from the detail shown in Figure 14, beside the Vis image (Figure 14a), it can be observed that photogrammetry (Figure 14b) reproduces all thickness variations, such as cracks, lacunae, and the thickness of painted areas. The normal map (Figure 14c) produces a very similar result but with an amplification of these variations: the horizontal papyrus fibers are much more evident, and the granularity of the Egyptian blue painted area is also significantly enhanced. Figure 15 shows another detail, which in this case was also acquired using VIL photogrammetry. For this comparison, the most accurate result from a morphological and metric standpoint is provided by visible-light photogrammetry (Figure 15b). The details visualized through RTI (Figure 15c) again represent more clearly the profiles of the horizontal papyrus fibers, as well as those of the decorative elements of the Figure’s garment and headdress. Finally, the photogrammetry performed in VIL (Figure 15d) shows artifacts when compared with VIS photogrammetry. The Egyptian blue area, which appears very bright in VIL, is represented in the 3D model not by increased thickness but by exaggerated relief relative to the surrounding contours, which are also quite noisy. This effect can be attributed to the lower signal-to-noise ratio of non-luminescent pixels in the VIL images.

**Figure 16:**
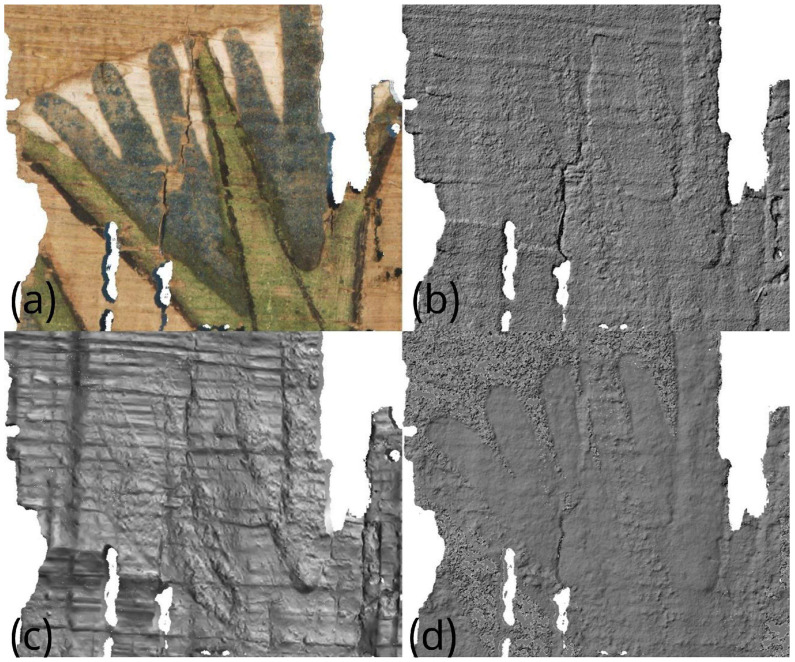
Comparison of the morphological representation of a papyrus detail obtained using: (**a**) visible-light image (VIS); (**b**) VIS ultra-close-range photogrammetry; (**c**) processed RTI normal map with flattened base plane; (**d**) VIL ultra-close-range photogrammetry.

A similar result is observed in the detail shown in Figure 16, where the fibers and painted areas of the lotus flower are represented differently depending on the technique employed. In this case as well, individual morphological features are variously emphasized according to the technique, resulting either in an increased apparent irregularity of the papyrus surface or in variations in the perceived thickness of the pictorial materials.

### 3.5. RTI and Multiband Photogrammetry: Quantitative Comparison

A quantitative and qualitative comparison between the data acquired via multiband UCRP and RTI coupled with normal map integration is detailed below and summarized in Table 2.

To capture the entire papyrus surface utilizing ultra-close-range photogrammetry, 464 images were acquired, generating a 490-million-point dense cloud and a 47-million-face mesh with a point density of 170 pts/mm^2^. Conversely, the RTI with normal map integration required only 94 images, producing a significantly lighter dataset: a 26-million-point cloud and a 5-million-face mesh at 11 pts/mm^2^. While the camera sensor pitch is comparable between the two methodologies (4.87 µm/px for photogrammetry vs. 4.63 µm/px for RTI), the Ground Sampling Distance (GSD) in object space is finer than in the ultra-close-range setup (17 µm/px vs. 25 µm/px). Acquisition and processing times further accentuate the methodological differences: photogrammetry required approximately 1 h of image capture followed by 3 days of computational processing, whereas RTI required only 10 min of capture and 30 min of processing.

The surface density (170 vs. 11 pts/mm^2^) confirms that ultra-close-range photogrammetry delivers a vastly richer 3D spatial dataset. To evaluate the macroscopic surface deviation of each standalone model, a best-fit idealized reference plane was computed for each point cloud, followed by the generation of a Digital Elevation Model (DEM) with a 10 µm grid resolution. Statistical analysis of the resulting height grid values reveals that the photogrammetric model exhibits a higher standard deviation (0.000495 m vs. 0.000244 m) and Root Mean Square (RMS) deviation (0.00302 m vs. 0.00252 m) compared to the RTI method. Rather than indicating higher sensor noise, these higher deviation values in the photogrammetric dataset are a direct result of its superior spatial resolution (170 pts/mm^2^), which successfully captures the true macroscopic morphological undulations and structural deformations of the papyrus surface. In contrast, the lower deviation values of the RTI dataset result from its lower geometric resolution, which produces a smoother, flatter macroscopic mesh that more closely aligns with a theoretical flat plane, despite its enhanced ability to visually render high-frequency fiber details via normal mapping.

To quantitatively assess the spatial variations between the two datasets, the Multiscale Model to Model Cloud Comparison (M3C2) [59] algorithm was employed, utilizing the standard photogrammetric point cloud as the reference model. This approach mitigates the influence of varying point densities and computes robust local distances along the surface normals. The statistical analysis of the M3C2 distance field yielded a near-zero Mean Deviation (µ) of 0.000015 m and a Standard Deviation (σ) of 0.000478 m.

The near-zero mean value indicates an excellent global co-registration between the two datasets. However, an analysis of the spatial distribution of the computed M3C2 distances reveals continuous, low-frequency morphological variations, with peak absolute deviations reaching ±0.002 m (2 mm). This spatial pattern strongly suggests the presence of a slight systematic deformation—attributable to algorithmic constraints during the 3D surface reconstruction—rather than localized variations in the capture of micro-topographic surface details. Despite this sub-millimetric global discrepancy, qualitative assessments demonstrate that the RTI approach significantly enhances the readability of high-frequency surface features (such as fiber visibility and interactive relighting), offering an optimal balance between operational efficiency and micro-detail enhancement, as highlighted in Table 2.

## 4. Conclusions

This study demonstrates the feasibility of applying three-dimensional acquisition and analysis techniques to artifacts conventionally treated as two-dimensional, such as papyri, for which analytical investigations are typically limited to textual readability or material characterization. From a conservation and diagnostic perspective, however, the three-dimensional geometry and surface morphology of papyrus represent critical parameters. In this context, photogrammetry and Reflectance Transformation Imaging (RTI) provide complementary approaches for the acquisition and documentation of spatial and volumetric information.

Due to the intrinsic material properties of papyrus, having small fragments, fiber separation and cracks, high spatial sampling is required to obtain a model with a high point-cloud density, fine spatial resolution, and detailed morphometric information. Quantitative analyses confirmed that UCRP represents the most robust solution for this purpose, enabling the acquisition of metrically reliable 3D models that accurately capture the true macroscopic morphological undulations of the artifact, including spectral bands beyond the visible range. Nonetheless, macro-photogrammetry is computationally and operationally demanding, requiring extended acquisition times and intensive processing workflows.

To address these limitations, RTI was investigated as an alternative, more efficient technique. Although RTI is intrinsically two-dimensional, the integration of RTI-derived normal maps allows the reconstruction of pseudo-3D surface models. Such reconstructions are affected by systematic low-frequency distortions (such as the doming effect observed in the M3C2 analysis) arising from the limitations of the normal integration process; however, the implementation of dedicated correction procedures enables the generation of surface models that are metrically comparable to those obtained via UCRP, while significantly reducing both acquisition and computation times.

Further improvements were achieved through the development of a custom MATLAB-based processing pipeline. This algorithm seamlessly fuses high-spatial-frequency information from RTI with low-spatial-frequency geometry derived from a low-resolution photogrammetric reference. The core of this integration relies on Fast Fourier Transform (FFT) blending in the frequency domain; notably, the effectiveness and final quality of this fusion are highly dependent on the user-defined radial thresholds (r_min_ and r_max_).

Following the frequency fusion, the pipeline integrates automated background masking and a customized spatial alignment utilizing k-nearest neighbors (k-NN) and Iterative Closest Point (ICP) algorithms, and finally, the hybrid point cloud is seamlessly re-imported into the photogrammetry software for full 3D texturing.

Our quantitative analyses demonstrate that this hybrid strategy exhibits minimal metric error when compared to the computationally heavy UCRP baseline. This proves that a fast, low-resolution photogrammetric survey, when coupled with our custom RTI integration code, is sufficient to obtain metrically comparable, high-fidelity micro-topographic results.

Overall, the proposed methodology offers a balanced compromise between geometric accuracy, spatial resolution, and computational complexity, making it particularly suitable for large-scale digital heritage applications. By reducing acquisition and processing costs while maintaining high metric reliability, this approach facilitates efficient data production, analysis, and dissemination for museums and cultural heritage institutions engaged in the digital documentation of fragile artifacts.

This research clearly indicates that the synergistic use of UCRP and RTI-derived models is an efficient tool to overcome the challenges associated with conventional 2D imaging. This integrated technique allows to determine texture, materials, micro-morphology, and conservation details that are usually difficult to assess from a 2D image or a single traditional 3D acquisition method.

## Figures and Tables

**Table 1:** Images and details of the fragments considered in this study.

	Object ID	Period	Type of Text	Provenance	Dimension (Cm)	Language
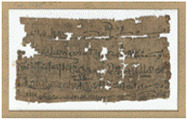	Cat. 1975	New Kingdom, 20th Dynasty (reign of Ramesses XI, 1106–1077 BCE)	Letter	Thebes, Deir el Medina (?)	12.0 × 22.3	Hieratic
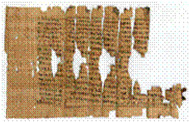	Cat. 2117; R. 08	Hellenistic Period (reign of Alexander the Great, 332–323 BCE)	Religious text, Book of Glorifications	Thebes	26.4 × 46.0	Hieratic
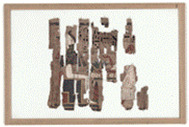	Provv. 6255	Third Intermediate Period, 21st Dynasty (1076–944 BCE)	Book of the Dead	Thebes (?)	22.0 × 24.0	Cursive Hieroglyphs
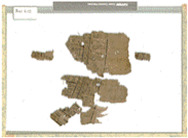	Provv. 6133	Ptolemaic Period (3rd–1st century BCE)	Template for coffin decoration	Unknown	6 fragments, ranging in size from 1.5 cm × 3.5 cm to 7.5 cm × 11 cm	Cursive Hieroglyphs
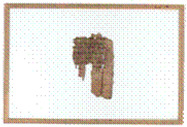	Suppl. 6101	Ptolemaic Period (3rd–1st century BCE)	Administrative document	Thebes, Deir el Medina	15.0 × 9.5	Demotic
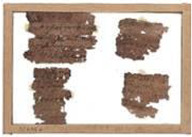	Provv. 8571; F 399/2	Byzantine Period (6th–7th century CE)	Administrative document	Unknown	4 fragments, different sizes	Coptic

**Table 2:** Data comparison acquired with ultra-close-range photogrammetry and with RTI Normal map integration.

Parameter	Ultra-Close-Range Photogrammetry	RTI Normal Map Int.	Advantage
**Acquisition Parameters**			
Number of Images	464	94	RTI (5×)
Camera Pixel Size	4.87 µm	4.63 µm	RTI
Ground Sampling Distance (GSD)	17 µm/px	25 µm/px	Photo (1.5×)
Acquisition Time	~1 h	~10 min	RTI (6×)
**Geometric & Spatial Data**			
Dense Cloud Points	490 M points	26 M points	Photo (19×)
Mesh Faces	47 M faces	5 M faces	Photo (9.5×)
Point Density	170 pts/mm^2^	11 pts/mm^2^	Photo (15×)
**Macroscopic Surface Deviation (DEM)**			
Mean	0.002980 m	0.002510 m	-
Std Deviation	0.000495 m	0.000244 m	Photo (2×)
RMS	0.00302 m	0.00252 m	Photo (1.2×)
**Operational Efficiency**			
Processing Time	~3 days	~30 min	RTI (144×)
Storage Requirements	~35 GB	~2 GB	RTI (17×)
**Qualitative Assessment**			
Quantitative Measurements	High precision	Moderate	Photo
Fiber Visibility	Standard	Enhanced (4–5×)	RTI
Interactive Relighting	No	Yes	RTI

## Data Availability

The raw data supporting the conclusions of this article will be made available by the authors on request.

## Abbreviations

The following abbreviations are used in this manuscript:

UCRP	Ultra-close-range photogrammetry
URCMP	Ultra-Close-Range Multiband Photogrammetry
RTI	Reflectance Transformation Imaging
GSD	Ground Sample Distance
DEM	Digital Elevation Model
Vis/RGB	Visible
NIR	Near-infrared reflected
UVF	UV-induced visible fluorescence
VIL	Visible-induced infrared luminescence
IRFC	Infrared False Color
PSD	Power Spectral Density
SfM	Structure from Motion
MBI	Multiband imaging
Gpx	Gigapixels
k-NN	k-nearest neighbors
ICP	Iterative Closest Point
RMS	Root Mean Square
